# Evidence of horizontal gene transfer within *porB* in 19 018 whole-genome *Neisseria* spp. isolates: a global phylogenetic analysis

**DOI:** 10.1099/mgen.0.001041

**Published:** 2023-06-09

**Authors:** Sheeba Santhini Manoharan-Basil, Zina Gestels, Saïd Abdellati, Elvis Achondou Akomoneh, Chris Kenyon

**Affiliations:** ^1^​ HIV/STI Unit, Department of Clinical Sciences, Institute of Tropical Medicine, 2000 Antwerp, Belgium; ^2^​ Clinical Reference Laboratory, Department of Clinical Sciences, Institute of Tropical Medicine, 2000 Antwerp, Belgium; ^3^​ Department of Microbiology and Parasitology, University of Bamenda, Cameroon; ^4^​ Division of Infectious Diseases and HIV Medicine, University of Cape Town, Anzio Road, Observatory 7700, South Africa

**Keywords:** porin, PorB1a, Porb1b, *Neisseria*, phylogenetics, commensal *Neisseria*

## Abstract

The PorB porins are the major pore-forming proteins in the genus *Neisseria *. The trimeric PorB porins consist of 16 highly conserved transmembrane domains that form an amphipathic β-sheet connected by short periplasmic turns and eight extracellular hydrophilic loops. These loops are immunogenic and also play an important role in mediating antimicrobial influx. This study sought to (i) characterize the variations in Neisserial loop 3(355–438 bp) associated with intermediate resistance to penicillin/tetracycline and (ii) evaluate if there was evidence of horizontal gene transfer in any of the loops. We collated an integrated database consisting of 19 018 *Neisseria* spp. genomes – 17 882 *Neisseria gonorrhoeae *, 114 *Neisseria meningitidis* and 1022 commensal *Neisseria* spp. To identify the *porB* alleles, a gene-by-gene approach (chewBBACA) was employed. To evaluate the presence of recombination events, the Recombination Detection Programme (RDP4) was used. In total, 3885 *porB* alleles were detected. Paralogues were identified in 17 *Neisseria* isolates. Putative recombination was identified in loop regions. Intraspecies recombination among *N. gonorrhoeae* isolates and interspecies recombination between *N. meningitidis* and commensal *Neisseria* spp., and *N. gonorrhoeae* and *N. lactamica* were identified. Here, we present a large-scale study of 19 018 *Neisseria* isolates to describe recombination and variation in the porB gene. Importantly, we found putative recombination in loop regions between the pathogenic and non-pathogenic *Neisseria* spp. These findings suggest the need for pheno- and genotypic surveillance of antimicrobial susceptibility in commensal *Neisseria* spp. to prevent the emergence of AMR in the pathogenic *Neisseria *. This article contains data hosted by Microreact.

## Data Summary

All the data used in this study have been publicly sourced. Graphical created using Microreact is available via specified web links: https://microreact.org/project/3xqrzaAdwarJK8YnGwuzbi-neisserialporins. Supplementary figures and tables have been deposited in Figshare; https://doi.org/10.6084/m9.figshare.22691506.v1 [1].

All other relevant data are available from the corresponding author.

**Impact Statement**

The PorB porins are the major pore-forming proteins in the genus *Neisseria *. They play an important role in regulating the entry of antimicrobials such as beta-lactams and tetracyclines into the cytoplasm. The extracellular loops are important immunogens targeted by anti-gonococcal vaccines, and mutations in loop 3 of PorB have been associated with intermediate resistance to penicillin/tetracycline. In this study, we used a collection of 19 018 *Neisseria* spp. genomes to characterize the variations in loop 3 and evaluate if there was evidence of horizontal gene transfer in any of the extracellular loops. We found evidence of recombination in the loop regions. Intraspecies recombination among *N. gonorrhoeae* isolates, and interspecies recombination between *N. meningitidis* and commensal *Neisseria* spp., and *N. gonorrhoeae* and *N. lactamica* were identified. These findings suggest that the *Neisseria* commensal species serve as a reservoir of genetic variation that can be taken up by the pathogenic *Neisseria* species. This has important implications for the development of vaccines targeting PorB and the emergence of antimicrobial resistance. As a result, it may be prudent to include genotypic surveillance of commensal *Neisseria* spp. in current surveillance programmes of pathogenic *Neisseria *.

## Introduction

The genus *Neisseria* comprises both pathogenic and non-pathogenic species. The pathogenic species, *Neisseria gonorrhoeae* and *Neisseria meningitidis *, predominantly colonize the human ano-genital and respiratory tracts [2–4]. Commensal *Neisseria* spp. are part of the normal flora of the upper respiratory tract, although certain species, such as *N. sicca *, *N. subflava *, *N. cinerea* and *N. lactamica *, occasionally cause invasive infections in humans. These infections include meningitis, endocarditis, ocular infections, pericarditis, empyema, peritonitis, septic arthritis, bursitis and osteomyelitis that were primarily based on individual case reports [5–10].

Porins are trimeric β-barrel membrane proteins composed of 35 kDa monomers with a 16-strand β-barrel fold and eight surface-exposed, variable, hydrophilic loops [11, 12]. Porins cross the cell membrane and act as a pore through which molecules, such as nutrients, toxins and antibiotics, can diffuse [13, 14]. Neisserial porins not only serve as channels through which water and solutes of less than 1000 MW can diffuse through the outer membrane, but also play an active role in pathogenesis and antibiotic resistance [15, 16]. Neisserial porins share sequence homology in the transmembrane domains, but the sequences of extracellular loops (1 through 8) have a high degree of variability between species and strains [17, 18].

All *Neisseria* spp. express porins, and these porins are the most represented outer-membrane protein [19]. These porins play an important role in virulence, immune evasion and resistance [20]. Most *Neisseria* species express one porin (Por) [11]. *N. meningitidis* is exceptional because it contains two por loci and expresses two porins, PorA and PorB [21]. Whilst, *N. gonorrhoeae* has both *porA* and *porB* genes that encodes PIA and PIB, respectively; the *porA* gene is not expressed due to frameshift and promoter mutations [11, 20, 22]. Gonococcal *porB* exists in one of two allelic forms, *porB1a* and *porB1b* [23–25]. Gonococcal *porB1a* isolates have an increased capacity for invasion of the bloodstream and causing disseminated infections, while *porB1b* isolates may be more likely to result in ascending infection of fallopian tubes [26, 27]. Alterations, modification and reduction in the expression of porins have all been associated with antimicrobial resistance (AMR) [19, 20]. *PenB* (encodes altered forms of PIB) mutations coding for amino acid (aa) positions 120 and 121 (of loop 3 that forms the pore constriction zone of PIB) result in decreased membrane permeability and hence decreased uptake of cephalosporins, penicillins and tetracyclines [20, 28, 29]. These *penB* mutations are an important cause of reduced susceptibility to these classes of antimicrobials [20].

Neisserial porins likely play an important role in immune evasion [20]. Antigenic variation of the gonococcal porins remains one of the challenges of vaccine development [30–32]. Little is known about the antigenic diversity of the commensal *Neisseria* and the antigenic relationships between non-pathogenic and pathogenic *Neisseria* species with the exception of *N. lactamica* and *N. meningitidis* [33–35].

*Neisseria* is naturally competent for the uptake of DNA. Horizontal gene transfer (HGT) of *penA*, *mtrCDE, rpsE*, *rplD, gyrA, gyrB* and *parE* from commensal *Neisseria* has been shown to be important in the genesis of resistance to β-lactams, macrolides and fluoroquinolones in the pathogenic *Neisseria* [36–42].

Previous studies in *Escherichia coli* and *Yersinia* spp. have found evidence of extensive intra- and interspecies recombination in the genes coding for the surface exposed regions of OmpF and other major porin proteins [43, 44]. Less is known about the extent of HGT in the Neisserial porins and their role in antigenic variation and AMR. A limited number of studies have characterized HGT and antigenic variation in the genus *Neisseria *. Frequent intraspecies recombination among gonococcal housekeeping genes and hypervariable *porB* and *tbpB* have been described [45, 46]. Recombination in a collection of 204 isolates collected in a Baltimore STI clinic over a decade showed a higher recombination rate in PIB than PIA and 13 housekeeping genes [47]. In the mature PorB protein, the mutability of 328 aa were examined and found that 308 aa were likely to be mutable and 20 aa were likely to be nonmutable [48]. One important paper found evidence of recombination in the surface loops of *porB* of *N. meningitidis* [49]. This analysis was, however, limited to 35 alleles of *porB*. Another phylogenetic analysis suggested evidence of horizontal gene transfer in the evolution of different porin classes and confirmed the close evolutionary relationships of the porins from *N. meningitidis *, *N. gonorrhoeae *, *N. lactamica* and *N. polysaccharea* [11].

Recombination analyses rely on contrasting evolutionary histories in different parts of the DNA sequences, leading to incongruence between gene trees at individual loci or mosaic structures within gene sequences or measurements of linkage disequilibrium between loci [50–54]. Recombination can be detected using multiple techniques [52, 55]. In this study, we sought to detect recombination in *porB* using the Recombination Detection Programme (RDP4) and by inferring incongruence in the *porB* gene tree in a population of *N. gonorrhoeae* and other *Neisseria* isolates using a dataset of 19 018 globally sourced *Neisseria* isolates [52, 56]. We determined the variations in loop 3(355–438 bp) at amino acid positions G120 and A121 associated with intermediate resistance to penicillin/tetracycline.

## Methods

### Genome collection

Whole-genome sequences (WGS) and metadata were downloaded from pubMLST [57] and pathogenwatch (https://pathogen.watch/ngonorrhoeae) from 86 countries in July 2022. This resulted in an integrated database containing WGS from 19 018 isolates, including 17 882 from *N. gonorrhoeae,* 114 *N *. *meningitidis* (the global data set [58], 625 *N *. *lactamica* and 397 other *Neisseria* spp. (When referring to species in plural spp. is used.) The numbers of isolates per species used in this study are listed in Table S1, available in the online version of this article. The quality of the genomes analysed using Quality Assessment Tool for Genome Assemblies (QUAST) [59] is provided in Table S2.

### Allele calling

Allele calling analysis was carried out as described in [38, 39] (Fig. S1). In brief, a study-specific schema was created. WGS from 19 018 isolates were analysed using chewBBACA software v.3.1.0 [60], followed by recombination analysis using the recombination detection programme, RDP4 v.4.100 [52]. For the chewBBACA analysis, a training dataset was created from the complete reference genome sequence of *N. gonorrhoeae* FA1090 using Prodigal v2.6.3 [61]. As the vast majority of genomes used in the study included gonococcal genomes, *N. gonorrhoeae* FA1090 was used as the reference genome. FA1090 was selected as the reference strain as it is a commonly used reference strain, has been used in the development of vaccine prototypes and carries antigen sequence types identical to the most broadly distributed antigen variants [30, 62, 63]. This was followed by creating a study-specific *Neisseria* scheme from 11 complete *Neisseria* genomes [* N. cinerea* (NZ_LS483369.1), *N. elongata* (NZ_CP007726.1), *N. meningitidis* (AE002098.2), *N. polysacchareae* (CP031325), *N. mucosa* (CP053939), *N. gonorrhoeae* FA1090 (NZ_CP115654.1) and MS11 (CP078118.1), *N. subflava* (NZ_CP039887.1), *N. lactamica* (NZ_CP031253.1), *N. flavescens* (NZ_CP039886), *N. sicca* (NZ_CP072524.1)]. Then the FASTA file for each coding sequence (CDS) was generated, followed by the creation of whole-genome (wg) multi-locus sequence typing (MLST) loci. The core-genome (cg) MLST loci with the threshold of 0.95, i.e. loci present in >95 % of the genomes, were then extracted from the wgMLST loci and visualized using a grape tree; the clusters isolate based on their allelic profiles using a minimum spanning algorithm [64]. Finally, the functional information of the CDS was retrieved using UniProtFinder (https://www.uniprot.org/). Multiple sequences were aligned using mafft v7.515, and neighbour-joining (NJ) trees were created using SchemaEvaluator implemented in chewBBACA [65]. *PorB* was identified based on the UniProt identifier, and the aligned sequences and NJ trees were exported from the SchemaEvaluator. The multiple sequence alignment files were imported into the molecular evolutionary genetic analysis tool, mega X, and the CDS was translated [66]. The NJ trees and the corresponding metadata were visualized using iTOL [67].

### Identification of *porB1a* and *porB1b* genes and substitutions at amino acid positions 120 and 121

Gonococci can be divided into serogroups WI, WII and WIII by coagglutination [68]. In the current study, the strains were assigned to PorB1a (WI) or PorB1b (WII/III) serogroups [63, 68]. The following search was conducted to classify the PorB1a and PorB1b serogroups in the *porB* FASTA sequences generated during the allele calling: Firstly, a search against NCBI protein databases with the term ‘trimeric protein PorB.IA’ and ‘trimeric protein PorB.IB’ retrieved 41 and 411 sequences, respectively (accessed date: March 2022). Secondly, a local blastp database of the PorB.IA and PorB.IB sequences were created in CLC Genomics Workbench (v 20.1.2). Lastly, a blastp was carried out against the PorB allele FASTA files, and sequences with 100 % identity were classified as belonging to either PorB1a or PorB1b serogroups

*N. gonorrhoeae* sequence typing for antimicrobial resistance (NG-STAR) uses a small 60 bp sequence that includes the G120- and A121-encoding region associated with antimicrobial resistance to limit the number of alleles of the hypervariable *porB* gene [69]. Due to the high homology of PorB1a sequences, the G120/A121 mutations apply only to PorB1b. However, both genes are included in NG-STAR to enhance typing purposes [69]. Therefore, in the present study, amino acid (aa) changes at positions 120 and 121 for both PorB1a and PorB1b were included.

The *porB* sequences extracted from the SchemaEvaluator were imported to CLC Genomics Workbench v 20.1.2. The nucleotide sequences were translated to protein sequences and aligned using mafft [65]. G120 and A121 aa positions were deduced based on the amino acid sequence ARO:3 000 464, retrieved from the CARD database [70].

### Phylogenetic and recombination analyses

Phylogenetic analysis was carried out on all the *porB* alleles representing 19 018 *Neisseria* isolates and a subclade comprising the PorB1a serogroup clustered together with *N. meningitidis* and *Neisseria* commensals, representative of 2653 isolates.

The nucleotide alignment of *porB* alleles (*n*=3,885) and the subclade (*n*=698) were screened for recombination events using the Recombination Detection Programme (RDP4) [52]. The analysis was carried out as described in [38, 39]. In brief, RDP4 examines the nucleotide sequence alignments and identifies recombinant events using recombination detection methods such as RDP, GENECONV, Bootscan, Maxchi, Chimaera, SiSscan and 3Seq [71–75]. Recombinant events supported by at least two of the seven algorithms were used with default settings, except the window size was increased to 60 nt in RDP, 120 nt in MaxChi and Chimaera, and to 500 in BootScan and SiScan. NJ trees were constructed using 1000 bootstrap replicates [76]. The minor parent and major parent were defined as the ones contributing to the smaller and larger fraction of the recombinant, respectively [52].

### Statistical analysis

The European Committee on Antimicrobial Susceptibility Testing (eucast) breakpoints v 12.0, 2022 (http://www.eucast.org/clinical_breakpoints/) were used to define penicillin and tetracycline MICs. The MICs of penicillin to susceptible (*S*), intermediate (*I*), resistant (*R*), and high-level resistant (HLR) *N. gonorrhoeae* were <=0.06 mg l^−1^, >0.06 to <=1 mg l^−1^, >1 to <128 mg l^−1^, >=128 mg l^−1^, respectively [77]. The MIC of tetracycline to *S*/*I*/*R*/HLR *N. gonorrhoeae* were <=0.5 mg l^−1^, >0.5 mg l^−1^ to <1 mg l^−1^, >1 to 16 mg l^−1^, and >16 mg l^−1^, respectively [78]. For *N. gonorrhoeae* isolates, the dataset with MICs that were reported only as *S*/*I*/*R*/HLR; the geometric mean (GM) MICs were used. In other words, an *N. gonorrhoeae* isolate with '*S*' for penicillin was given the value of 0.03 mg l^−1^ by calculating the GM for all susceptible isolates with the MIC value. This process resulted in the penicillin *S*/*I*/*R*/HLR results being changed to 0.03 mg l^−1^, 0.57 mg l^−1^, 4.1 mg l^−1^ and 217.3 mg l^−1^, respectively. Isolates with tetracycline MICs of *S*/*I*/*R*/HLR were transformed to 0.31 mg l^−1^, 0.75 mg l^−1^, 6.1 mg l^−1^ and 39.8 mg l^−1^, respectively.

Statistical analyses, including the calculation of GMs, were performed using XLSTAT (2022, Statistical and data analysis solution, New York, USA). MIC variables were log_2_-transformed for use as continuous outcome variables [39]. To evaluate the correlation between multiple amino acid substitutions and to assess the correlations between phenotypic and genotypic patterns of resistance to penicillin and tetracycline, the two-sided Mann–Whitney *U* test was used. A value of *P* of <0.001 was considered statistically significant.

## Results

### *porB* alleles

**Fig. 1.**
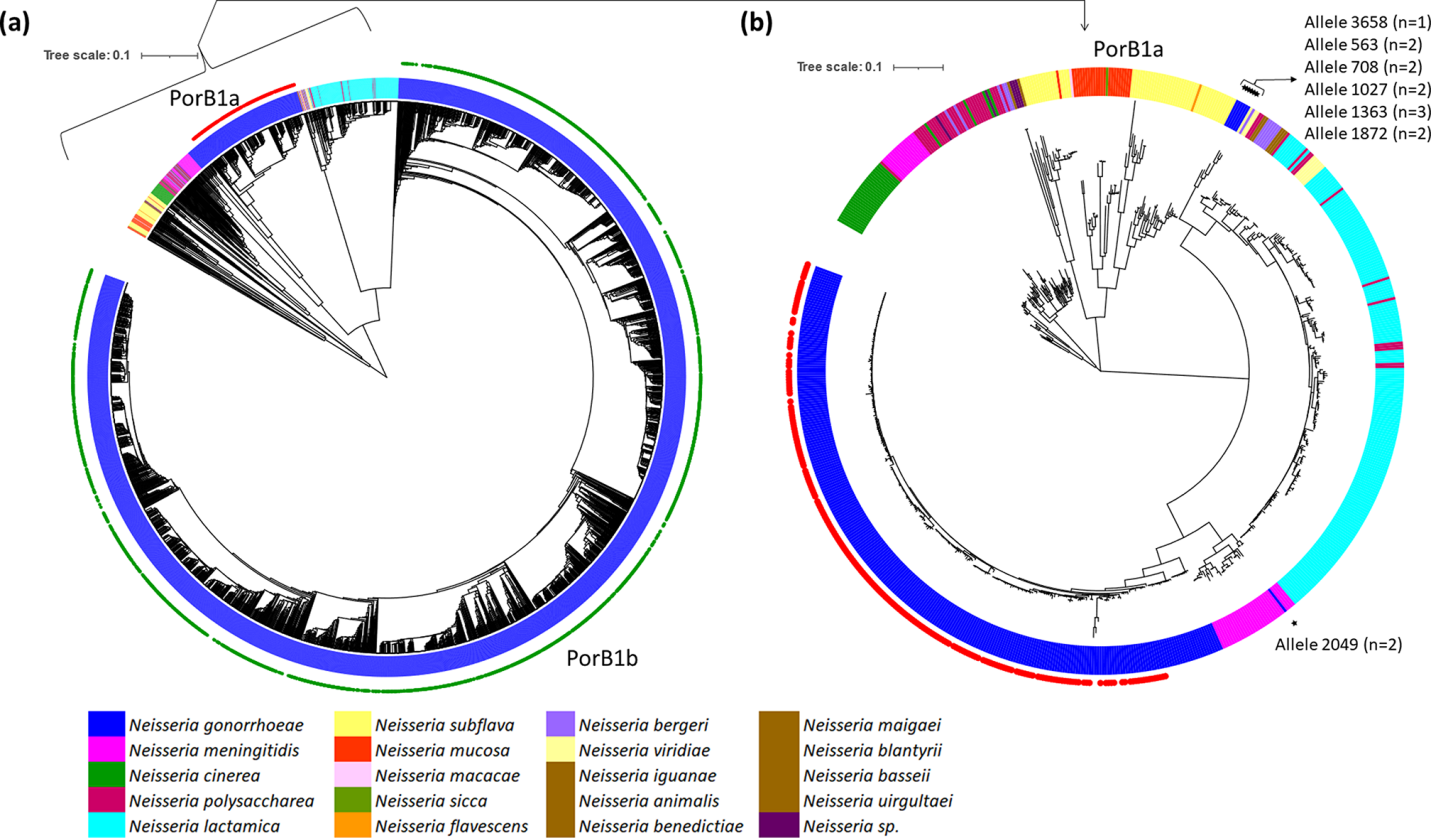
(**a**) Phylogenetic tree of *porB* alleles (*n*=3885) (**b**) Phylogenetic tree of PorB1a allele clade (*n*=698). The figure was generated using iTOL. Blue, magenta, cyan and yellow colour nodes denotes *N. gonorrhoeae, N. meningitidis*, *N. lactamica* and *N. subflava,* respectively, and the other colours denote other *Neisseria* spp. Red and green filled circles denote the porB1a and porB1b alleles, respectively. Black stars denote evidence of horizontal gene transfer.

A total of 3885 *porB* alleles were identified in the *Neisseria* spp. (Fig. 1a). Eighty-nine percent (3,461/3,885) of the *porB* alleles were found in *N. gonorrhoeae *, 4.7 %(185/3 885) in *N. lactamica *, 1.4 %(58/3 885) in *N.subflava*, 1.2 %(47/3 885) in *N. meningitidis *, 1.1 %(44/3 885) in *N. polysaccharea *, 0.7 %(31/3 885) in *N. cinerea *, 0.5 %(23/3 885) in *N. mucosa *, 0.3 %(12/3 885) in *N. bergeri*, and 0.2 %(9/3 885) in *N. viridiae*. The rest of the *Neisseria* species (*n*=10) were represented by unique *porB* alleles (Fig. S2). The *porB* locus was present in 99.7 %(17 831/17 882) of *N. gonorrhoeae* isolates, 84.2 %(96/114) of *N. meningitidis* and 93.2 %(953/1,022) of other *Neisseria* spp. Paralogues were identified in 0.08 %(17/19 018) *Neisseria* isolates. Non-informative paralogous hits (NIPH), i.e. alleles of the same locus with <100 % identity, were identified in six isolates [* N. cinerea* (*n*=2), *N. meningitidis* (*n*=3), *N. subflava* (*n*=1)]. NIPH exact match (NIPHEM), i.e. alleles of the same locus with 100 % identity, were identified in 11 isolates [* N. gonorrhoeae* (*n*=6), *N. meningitidis* (*n*=2), *N. subflava* (*n*=2)] *N. lactamica* (*n*=1)].

### Determination of PorB1a, PorB1b serotypes and geographical distribution of *porB* in *N. gonorrhoeae* isolates

**Fig. 2.**
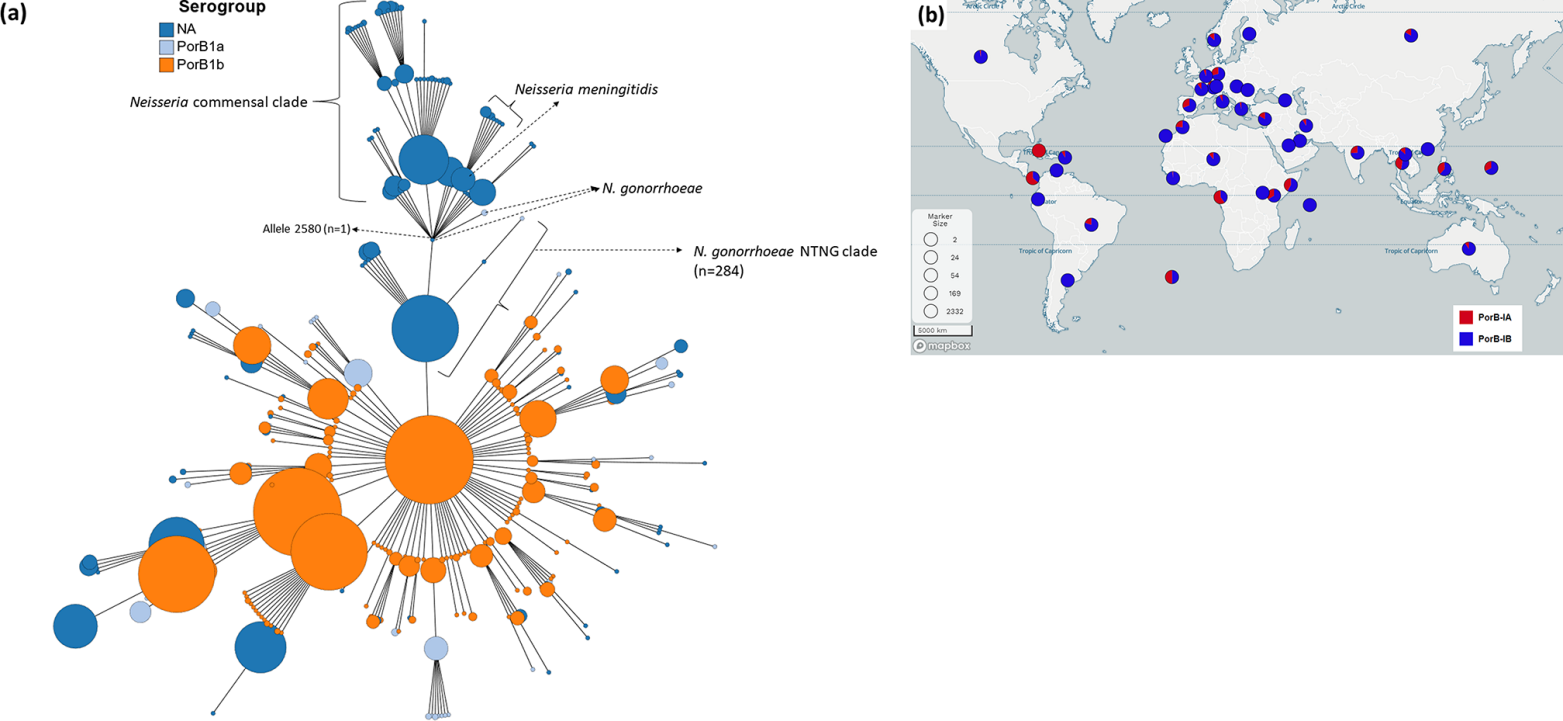
(**a**) Minimum spanning tree comparing core-genome allelic profiles in association with serogroups. (**b**) Global distribution of serogroups. Percentage of number of isolates is given in brackets. Isolates are displayed as circles. The size of each circle indicates the number of isolates of this particular type. Red and blue nodes denotes the PorB1a and PorB1B serogroup, respectively. Interactive map is available at https://microreact.org/project/3xqrzaAdwarJK8YnGwuzbi-neisserialporins. NTNG – non-typeable *N. gonorrhoeae *.

The 3461 *porB* alleles identified in *N. gonorrhoeae* were further classified as either belonging to PorB1a or PorB1b serogroup. Of the 17, 831 *N *. *gonorrhoeae* isolates that contained a *porB* allele, 8 %(1 441/17 831) and 78 %(13 915/17 831) were found to possess *porB1a* and *porB1b* genes, respectively (Fig. 1a, b). In total, 13.8 % of isolates could not be assigned to either of the above serogroups (2 475/17 831), Fig. 2a.

The PorB1a and PorB1b serogroups were spread across 47 and 67 countries, respectively (Fig. 2b, Table S3). The individual variants did not appear to be equally distributed worldwide (Fig. 2b, Table S3). For example, 302 isolates assigned to allele 835 belonging to the porB1a serogroup were spread worldwide (Americas, Australia and Europe). In contrast, 120 of the 122 isolates from allele 19 (PorB1b) were identified in the UK and no geographical data was available for the other two isolates (Fig. 2b, Table S3).

### Variations in loop 3 and antimicrobial susceptibility in *N. gonorrhoeae*

The surface-exposed loops (loops 1 to 8) were analysed individually, and the size of the loops varied for the *Neisseria* spp. as follows: (1) loop 1 — 19–35 aa, (2) loop 2 — 13–14 aa, (3) loop 3 — 24–32 aa, (4) loop 4 — 8–21 aa, (5) loop 5 — 9–37 aa, (6) loop 4 — 12–49 aa, (7) loop 7 — 12–16 aa and (8) loop 8— 8–14 aa (Fig. S3). Loop 3, which plays an important role in antigenic variability and antimicrobial susceptibility in *N. gonorrhoeae* was dissimilar to the corresponding loops in the commensal *Neisseria* spp., with rarely identical amino acid sequences and with no alleles shared (Figs 2 and S3).

**Fig. 3.**
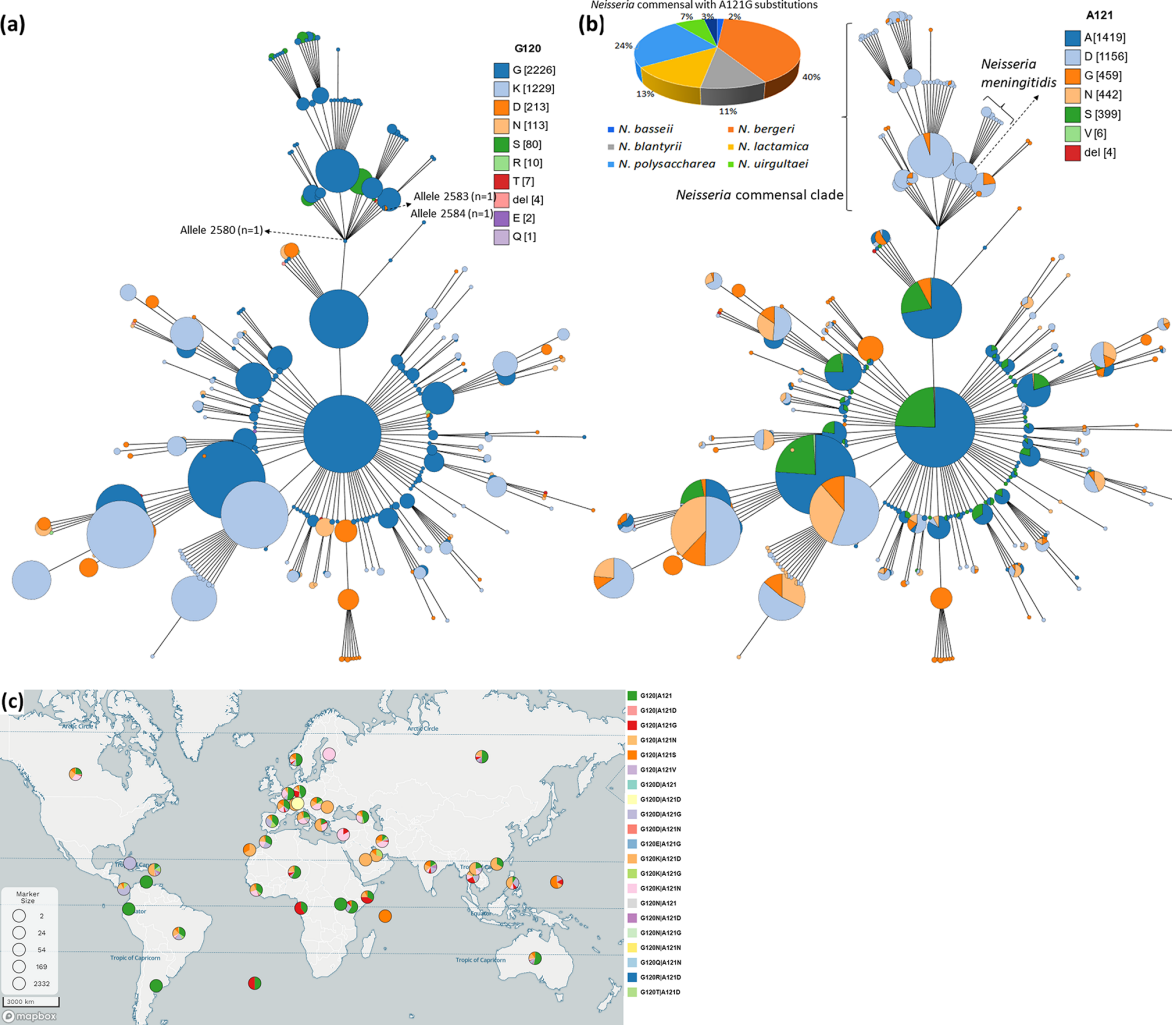
Minimum spanning tree comparing core-genome allelic profiles in association with substitutions at (**a**) G120 and (**b**) A121 amino acid positions. (**c**) Global distribution of substitutions. Percentage of number of isolates is given in brackets. Isolates are displayed as circles. Inset – percentage of *Neisseria* spp. with A121G substitution. The size of each circle indicates the number of isolates of a particular type. Interactive map is available at https://microreact.org/project/3xqrzaAdwarJK8YnGwuzbi-neisserialporins

Substitutions in 120 and 121 aa positions that result in reduced susceptibility to penicillin and tetracycline were deduced from the PorB protein sequences. Amino acid positions 120 and 121 corresponded to amino acid positions 172 and 173 in the full-length sequence alignment of all the *Neisseria* spp., and to amino acid positions 2 and 3 in loop 3, respectively (Fig. S3). G120 and A121 substitutions were detected in 17 882 isolates and were distributed worldwide (Fig. 3a–c). Penicillin and tetracycline MICs were available for 35.1 % of (6 283/17 882) and 25.9 %(4 639/17 882) isolates, respectively.

### PorB1b isolates

**Fig. 4.**
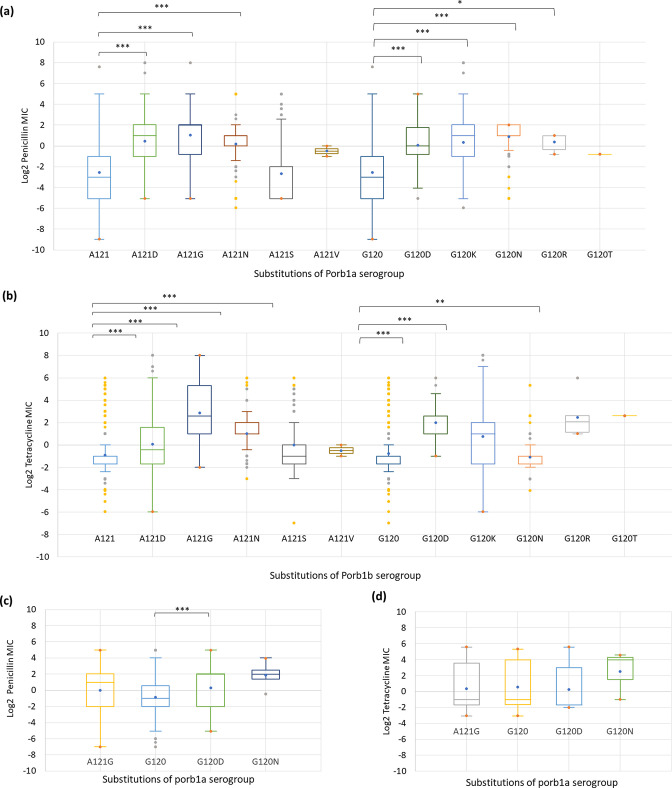
MIC distribution of (**a, c**) penicillin and (b, d) tetracycline for G120/A121 variants of porb1a and porb1b serogroups, respectively. Box plots and statistical analyses for all the isolates with MIC are shown. Statistical significance between variants and MIC distributions was assessed by Mann–Whitney *U* est: *<*I>P*<0.01, **<*I>P*<0.001, ***<*I>P*<0.0001. Blue colour box plot denotes the wild type. The line inside the box marks the median. The upper and the lower hinges corresponds to the 25th and 75th percentiles.

The most common substitutions detected in the PorB1b isolates were G120K/A121D (22.06 %; 3 069/13 915), G120K/A121N (15.6 %; 2 180/13 915) and G120K/A121G (2.04 %; 284/13 915) double substitutions. G120N/A121D, G120N/A121G, G120N/A121N, G120R/A121D, G120R/A121G and G120T/A121D double substitutions were less commonly identified (Figs 3a and 4a) (Table 1). A121D (0.1 %; 14/13 915), A121N (0.06 %; 8/13 915) and A121S (12.54 %; 1 745/13 915) single substitutions were identified.

**Table 1.** Mutations at the amino acid residues G120 and A121 of PorB

Substitutions	No. of PorB1a isolates (%)	No. of PorB1b isolates (%)	No. of PorB isolates (%)	No. of isolates with penicillin MIC (%)	No. of isolates with tetracycline MIC (%)
G120|A121	0	6044 (43.44)	7078 (39.58)	2973 (47.32)	2046 (44.1)
G120|A121D	0	14 (0.1)	18 (0.1)	5 (0.08)	4 (0.09)
G120|A121G	540 (37.4)	0 (0)	655 (3.66)	201 (3.2)	135 (2.91)
G120|A121N	0	8 (0.06)	8 (0.04)	5 (0.08)	2 (0.04)
G120|A121S	0	1745 (12.54)	2139 (11.96)	685 (10.9)	466 (10.05)
G120|A121V	0	11 (0.08)	12 (0.07)	2 (0.03)	2 (0.04)
G120D|A121	0	163 (1.17)	190 (1.06)	25 (0.4)	24 (0.52)
G120D|A121D	0	26 (0.19)	35 (0.2)	7 (0.11)	3 (0.06)
G120D|A121G	890 (61.76)	0 (0)	924 (5.17)	407 (6.48)	295 (6.36)
G120D|A121N	0	36 (0.26)	36 (0.2)	7 (0.11)	6 (0.13)
G120del|A121del	0	0 (0)	9 (0.05)	4 (0.06)	4 (0.09)
G120E|A121G	0	1 (0.01)	1 (0.01)	0 (0)	0 (0)
G120K|A121D	0	3069 (22.06)	3446 (19.27)	960 (15.28)	786 (16.94)
G120K|A121G	0	284 (2.04)	358 (2)	105 (1.67)	88 (1.9)
G120K|A121N	0	2180 (15.67)	2498 (13.97)	685 (10.9)	573 (12.35)
G120N|A121	0	19 (0.14)	31 (0.17)	8 (0.13)	7 (0.15)
G120N|A121D	0	237 (1.7)	278 (1.55)	164 (2.61)	164 (3.54)
G120N|A121G	11(0.76)	43 (0.31)	64 (0.36)	18 (0.29)	15 (0.32)
G120N|A121N	0	13 (0.09)	20 (0.11)	9 (0.14)	10 (0.22)
G120Q|A121N	0	2 (0.01)	2 (0.01)	0 (0)	0 (0)
G120R|A121D	0	17 (0.12)	20 (0.11)	6 (0.1)	6 (0.13)
G120R|A121G	0	0 (0)	1 (0.01)	1 (0.02)	1 (0.02)
G120T|A121D	0	3 (0.02)	8 (0.04)	2 (0.03)	2 (0.04)
na	0	0 (0)	51 (0.29)	4 (0.06)	0 (0)
Total	1441	13 915	17 882	6283	4639

Five substitutions were detected at aa position 120 in the Porb1b : G120D (0.3 %), G120K (14.8 %), G120N (1.8 %), G120R (0.06 %) and G120T (0.1 %). Substitutions G120D (*P*<0.0001), G120K (*P*<0.0001), G120N (*P*<0.0001), G120R (*P*<0.01) were associated with an increased penicillin MIC (Fig. 4c) and substitutions G120D (*P*<0.0001), G120K (*P*<0.0001) and G120R (*P*<0.001) were associated with increased tetracycline MICs (Fig. 4d). Five substitutions, A121D (10.6 %), A121G (1 %), A121N (5.1 %), A121S (6.1 %), A121V (0.02 %), were identified at aa position 121 in Porb1b and three of these substitutions (A121D, A121G and A121N) were associated with an increased penicillin MIC (*P*<0.0001, Fig. 4c). Four substitutions (A121D, A121G, A121N and A121S) were associated with increased tetracycline MICs (*P*<0.0001, Fig. 4d).

### PorB1a isolates

In the 1441 PorB1a *N. gonorrhoeae* isolates, 61.7 %(890/1 441) isolates had G120D/A121G double substitutions, 37.4 %(540/1 441) isolates had a single A121G single and 0.76 % (11/1,441) had G120N/A121G substitutions (Table 1).

Two substitutions, G120D (35 %) and G120N (0.3 %), were identified at aa position 120 in the Porb1a. G120D was significantly associated with an increased penicillin MIC (*P*<0.0001, Fig. 4a) when compared to G120 (wild-type, 13.7 %). No significant association between substitutions G120D (35.3 %), G120N (0.3 %), and an increase in tetracycline MIC were identified (Fig. 4b). Fifty percent of isolates had the penicillin and tetracycline MIC available for A121G substitution (Fig. 4a, b).

The following double substitutions were G120D/A121, G120D/A121G, G120K/A121D, G120K/A121G, G120K/A121N and G120N/A121D significantly associated with an increased penicillin MIC (*P*<0.0001, Figs 3a, 4a, c). G120/A121, G120D/A121G, G120K/A121D, G120K/A121G, G120K/A121N and A121S variants were significantly associated with an increased tetracycline MIC (*P*<0.0001, Figs 3a, 4c, d).

### Changes in the relative abundance of amino acid changes at positions 120 and 121 in *N. gonorrhoeae* serogroups over time

**Fig. 5.**
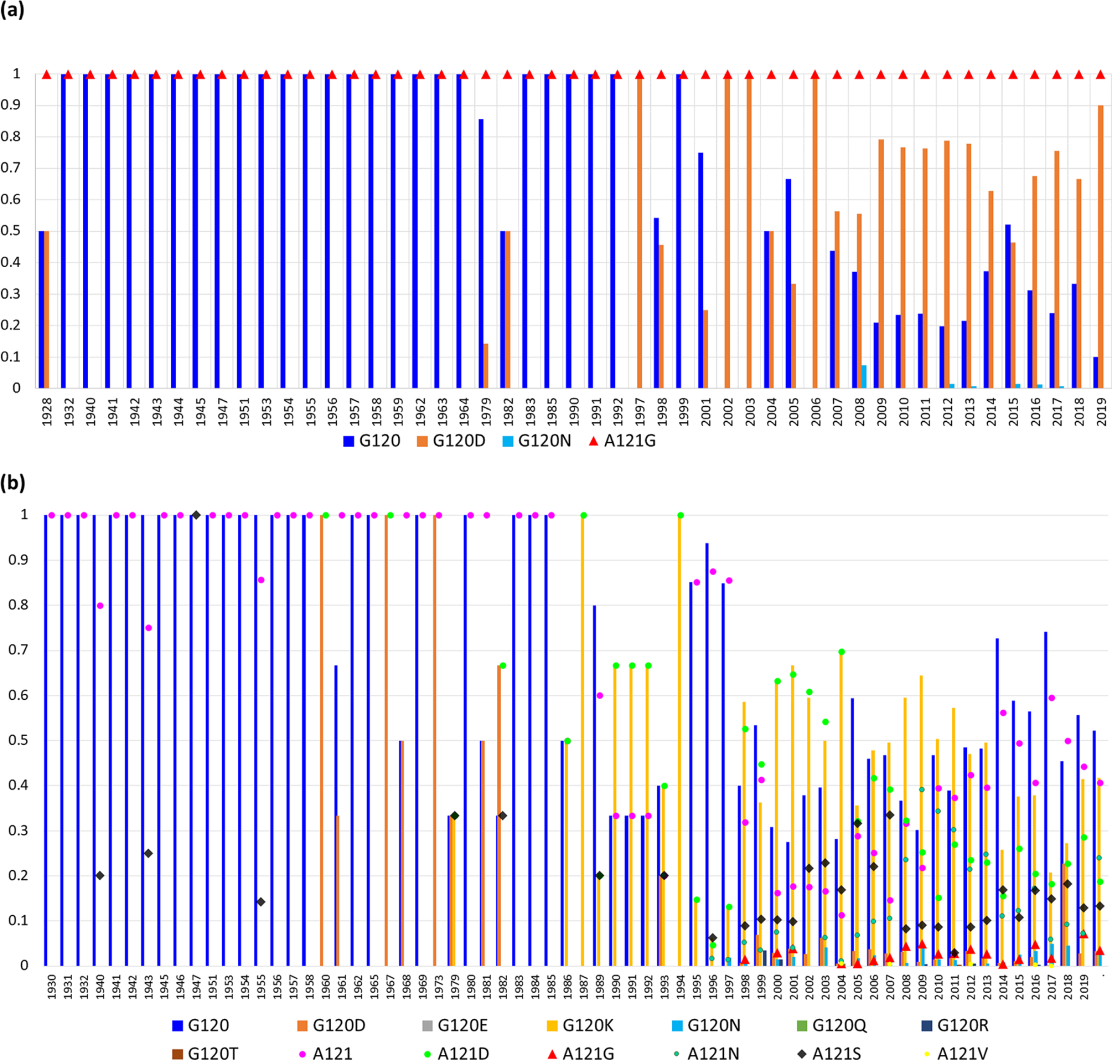
Time of emergence of resistance-associated mutations at positions 120 (columns) and 121 (scatter points) of (**a**) porB1a and (**b**) porb1b serogroups. *X* and *Y* axis denotes the year and the relative abundance of resistance-associated mutations in the *N. gonorrhoeae* isolates, respectively.

The *porB*-containing isolates were obtained between 1928 and 2019 (Fig. 5a, b, Table S4).

#### (i) Serogroup-Porb1a

Four isolates belonging to the Porb1a serogroup were available in 1928 (Fig. 5a). Out of the four isolates, two isolates each had both the G120D and A121G substitutions. Of note, the G120D substitution was first observed in 1928 and then not observed again until 1979. The G120N substitution was first observed in 2008. All the PorB1a isolates from 1928 to 2019 had a glycine at position 121.

#### (ii) Serogroup-Porb1b

Two isolates each from the year 1930 and 1931 and six isolates from 1932 were available (Fig. 5b). All ten isolates were wild-type (G120 and A121). The first G120 substitution, G120D was observed in 1960, and A121S substitution (*n*=2) was first observed in 1940.

G120K and A121D was first observed in 1979. Several other variants emerged around 1993. Of note, the G120Q/A121N (*n*=2), G120E/A121G (*n*=1), G120R/A121G (*n*=1) variants were only found in 2004, 2012 and 2017, respectively (Fig. 5b, STable S4).

### Evidence of horizontal gene transfer

RDP4 analysis and phylogenetic tree construction were carried out on (a) all the *porB* alleles (*n*=3,885) and (b) subclade consisting of the PorB1a serogroup clustered together with *N. meningitidis* and commensal *Neisseria* isolates (*n*=698).

#### (a) *porB* alleles (*n*=3885)

Twenty-seven unique recombination events were supported by at least two of seven detection methods using RDP4 analysis (Table S5). These included nine recombination events identified in the loop three regions. Intraspecies recombination among *N. gonorrhoeae* species and interspecies recombination were identified between *N. meningitidis* and commensal *Neisseria* spp.

All the recombinant events are summarized in Table S5, and an example of inter- and intra-species recombination is provided below:

(i) In event 9, *N. meningitidis* (allele—494; *n* = 2) was the recombinant. The breakpoint positions 130–622 in the recombinant *N. gonorrhoeae* sequence correspond to loops- L1 (91.158), L2 (244282), L3 (355.426), L4 (493.521) and L5 (592.633) in the alignment. The corresponding minor parent was *N. polysaccharea* (allele-979; *n* = 1; recombinant region—363–1027), and the major parent was *N. cinerea* (allele- 857; *n*=1; recombinant region 1–362 and 1028–2493 nt). Sequences with the same recombinant event were present in 16 alleles that consisted of 18 *N *. *meningitidis* (alleles-491, 492, 799, 805, 808, 813, 851, 861, 868, 1627, 1636)*,* three *N. polysaccharea* (alleles-7, 1152, 2563) and one *N. cinerea* (allele- 857) isolates. The event was supported by six methods (GENECONV, Bootscan, Maxchi, Chimaera, SiSscan and 3Seq). The minor, major parents and the recombinant had the A121D substitution.

(ii) Putative recombination event 1 with *N. gonorrhoeae* (allele-3223; *n*=1) as a recombinant was identified. The breakpoint positions 310–972 in the recombinant *N. gonorrhoeae* sequence correspond to L3 (355.426), L4 (493.521), L5 (592.633), L6 (703.747), L7 (820.858) and L8 (925.960) in the alignment. The event was supported by five methods (GENECONV, Bootscan, Maxchi, and SiSscan and 3Seq). Evidence of the same recombinant event was present in two other alleles belonging to *N. gonorrhoeae* isolates (allele-3805, *n*=1 and allele-2934; *n*=1). The corresponding minor parent was *N. gonorrhoeae* (allele-779; *n*=4; recombinant region – 593–2481 nt), and major parent was *N. gonorrhoeae* (allele-3654; *n*=1; recombinant region – 1–592 and 2482–2493 nt; Table S5). The minor parent, *N. gonorrhoeae* isolate, allele-779, had the G120D/A121G substitutions, and the major parent, *N. gonorrhoeae* isolates, allele-3654, had the G120N substitution. The recombinant *N. gonorrhoeae* isolates (alleles-3223; 3805 and 2934) had the A121G substitution (Table S5).

#### (b) Subclade consisting of PorB1a-type alleles belonging to *N. gonorrhoeae* and other alleles from other *Neisseria* spp.(*n*=698)

The region of the tree containing the subclade of porB1a alleles was clustered together across *N. meningitidis* and other *Neisseria* species (Fig. 1a, b). The subclade was further investigated using RDP4, and 36 unique recombination events were supported by at least two out of seven detection methods. Interspecies recombination was identified between *N. gonorrhoeae* and commensal *Neisseria* spp. All the recombinant events are summarized in Table S6, and an example is provided below:

In event 18, *N. gonorrhoeae* (allele-3658; *n*=1) was the recombinant. The breakpoint positions 764–945 in the recombinant *N. gonorrhoeae* sequence correspond to loops- L7 (820-858) and L8 (925-960) in the alignment. The corresponding minor parent was *N. gonorrhoeae* (allele-3058; *n*=1; recombinant region – 982–1286), and the major parent was *N. lactamica* (allele-1179; *n*=1; recombinant region 1–981 and 1287–1397 nt). The event was supported by four methods (GENECONV, Maxchi, Chimaera and 3Seq).

Further investigation of allele-3658 (ST12044) in the phylogenetic tree revealed five *N. gonorrhoeae* alleles [allele-563, ST11412 (*n*=2); allele-708, ST1901 (*n*=2); allele-1027, ST-not available (*n*=2); allele-1363, ST1579 (*n*=3); allele-1872, ST6721 (*n*=2)] belonging to the same clade (Fig. 1b). The serogroup of the above alleles could not be determined. The major parent was isolated from a woman and the minor parent from a man who has sex with men. In another instance of recombination, *N. gonorrhoeae* allele-2,049 (*n*=2) was identified within one of the clades of *N. meningitidis* (Fig. 1b).

### Clusters of *Neisseria* isolates with A121G substitution

Several *N. gonorrhoeae* isolates could not be assigned to either of the two serogroups and hence non-typeable (*n*=2,526; representative of 886 alleles). Out of the 2526 isolates of non-typeable *Neisseria gonorrhoeae* (NTNG), three isolates [allele-2,580 (*n*=1); allele 2583 (*n*=1); allele 2584 (*n*=1)], Figs 2a and 3a) were clustered with *N. meningitidis *, and *Neisseria* commensals. Out of these three isolates, two were typeable and belonged to the porb1a serogroup (Fig. 2a), and one isolate (allele-2583) had the G120D, A121G substitutions, whereas another isolate (allele-2584) had no substitution at position 120 and had the A121G substitution. NTNG allele-2580 is a wild-type with no changes at aa positions 120 and 121. Two hundred and eighty-four NTNG isolates were in close proximity to the *Neisseria* commensal clade (Figs 2a and 3b). Out of the 284 isolates, 27 isolates had the A121G substitution. The commensal clade contained either the A121D (*n*=819) or the A121G (*n*=129, Fig. 3b) or A121S (*n*=1) substitutions, Fig. 3b.

## Discussion

We found evidence of HGT in the *porB* gene in *N. gonorrhoeae* and *N. meningitidis.* Additionally, using an allele-calling approach, we evaluated the substitutions at amino acid positions 120 and 121 to assess decreased susceptibility to penicillin and tetracycline within a global collection of 17882 *N. gonorrhoeae* genomes.

Using a cgMLST approach, we identified 3885 *porB* alleles from 17 882 *N. gonorrhoeae *, 114 *N. meningitidis* and 1022 *Neisseria* spp. isolates from around the world. Out of the 3885 *porB* alleles, 3461 alleles were identified in 17 831 *N. gonorrhoeae* isolates. The gonococcal alleles were further classified into serogroups as follows: PorB1a-216 alleles (isolates=1,441) and PorB1b-2,359 alleles (isolates=13,915). The remaining 886 alleles comprising 2,526 NTNG isolates could not be assigned to either of the above serogroups.

Gonococcal PorB1a and PorB1b play a role in the intra-host dissemination of *N. gonorrhoeae* [79], and mutations at the 120 and 121 sites are associated with elevated penicillin and tetracycline MIC values [25, 28, 47]. The gonococcal PorB1b differs from the PorB1a in having more mutations in its sequence [25, 80]. Out of the 1441 PorB1a *N. gonorrhoeae* isolates, G120D (35 %) was significantly associated with an increased penicillin MIC (, Fig. 4a). Among the 13 915 PorB1b isolates, five substitutions for both aa positions 120 (G120D, G120K, G120N, G120R, and G120T) and 121 (A121D, A121G, A121N, A121S, A121V) were identified. Out of the five substitutions, four substitutions [G120D, G120K, G120N and G120R] were significantly associated with an increased penicillin MIC (Fig. 4c) and three substitutions [G120D, G120K and G120R were associated with increased susceptibility to tetracycline (Fig. 4d). For position 121, three substitutions, A121D, A121G, A121N and four substitutions, A121D, A121G, A121N and A121S, were associated with an increased penicillin MIC (Fig. 4c) and tetracycline MIC (Fig. 4d), respectively.

G120D, A121D, A121G, A121N and A12S have previously been found to be associated with reduced susceptibility to penicillin and tetracycline [20, 29, 81, 82]. Interestingly, two of four Porb1a isolates from 1928 had G120D/A121G (*n*=2, alleles-1835, ST11688) substitutions, i.e. before the introduction of penicillin. Isolates belonging to Porb1b serogroup were available from 1930 onwards, and no mutations were observed in the isolates from the years 1930–32. The A121G substitution was first observed in two isolates belonging to Porb1b serogroup from 1998. Interestingly, the first resistance-associated mutation at aa position 121 (A121S) was observed in two isolates belonging to PorB1b serogroup from 1940. Of note, few NTNG isolates (*n*=27) and *Neisseria* commensal isolates (*n*=129) had the A121G substitution (Fig. 3a, b). These NTNG isolates were not further investigated and are beyond the scope of the current study. Though G120 and A121 substitutions apply to only Porb1b, both Porb1a and Porb1b are used for typing purposes [69]. Therefore, resolving the amino acid wild-type at position 121 for PorB1a, i.e. G121 instead of A121, will help resolve the NTNG isolates.

The geographical distribution of *N. gonorrhoeae* isolates with PorB1a, and PorB1b genotypes have been studied in several countries/regions [83]. In the current study, allele 835, consisting of 302 isolates belonging to the PorB1a serogroup, was spread worldwide (Americas, Australia and Europe), Fig. 2b. The high rate of HGT observed in the gonococcus led to the assumption that limited structure would be evident in its populations [54]. Allele 9, consisting of 122 isolates belonging to the PorB1b serogroup, was identified only in the UK, and this is consistent with a semi-clonal population structure [84–87]

HGT among and within the hypervariable *porB* has been previously described [45]. We found evidence of intra- and inter-species recombination among the *Neisseria* species. In the interspecies recombination, DNA was acquired by *N. meningitidis* from two commensal *Neisseria* species: *N. cinerea* and *N. polysaccharea *.

Phylogenetic incongruence was observed in a subclade that consisted of the PorB1a serogroup in the *porB* gene tree, which could have arisen due to HGT. Since HGT significantly influences bacterial phylogenomic patterns, this subclade with apparent incongruence was further investigated [56]. A subclade consisting of 698 *porB* alleles was extracted, and a phylogenetic tree was constructed. The multiple sequence alignment was then analysed using RDP4. Interestingly, this approach provided evidence of interspecies recombination between *N. gonorrhoeae* (minor parent) and *Neisseria commensal N. lactamica (major parent).*

Caveats of our study include the following: only computational analysis was carried out based on the publicly available data, and variations and recombination events were determined only for the *porB* gene. Analysis of variations was accounted only for G120 and A 120 amino acid positions, the most frequent antimicrobial-resistant determinant found in PorB. Moreover, due to the lack of availability of MIC data for the commensals, the correlation of MICs between the parents and the recombinants could not be determined. Some of the Pathogenwatch MICs are estimated based on genotype using 485.toml (https://gitlab.com/cgps/pathogenwatch/amr-libraries/blob/0.0.17/485.toml) and the estimated MICs may differ slightly from MICs obtained via phenotypic testing [38]. Finally, the HGT events were not confirmed experimentally.

Antimicrobial resistance in *N. gonorrhoeae* and *N. meningitidis* frequently emerges in commensal *Neisseria* species and is transferred to the pathogenic *Neisseria* via natural transformation [36–42]. In the present study, recombination was seen in the hypervariable loop regions, including loop 3, where the resistance-associated determinants are present [20, 29, 82]. HGT in loops of *porB* is worrisome and suggests the need for geno- and phenotypic surveillance of antimicrobial susceptibility in commensal *Neisseria* spp. and a pan-*Neisseria approach* to prevent the further emergence of AMR in the pathogenic *Neisseria* [88]. Similar considerations apply to vaccine strategies that target PorB [89–93].
